# A twin study exploring the association between childhood emotional and behaviour problems and specific psychotic experiences in a community sample of adolescents

**DOI:** 10.1111/jcpp.12839

**Published:** 2017-11-03

**Authors:** Sania Shakoor, Philip McGuire, Alastair G. Cardno, Daniel Freeman, Angelica Ronald

**Affiliations:** ^1^ School of Law, Social and Behavioural Sciences University of Kingston Kingston upon Thames UK; ^2^ Department of Psychosis Studies Institute of Psychiatry, Psychology Neuroscience King's College London London UK; ^3^ Academic Unit of Psychiatry and Behavioural Sciences University of Leeds Leeds UK; ^4^ Department of Psychiatry University of Oxford Oxford UK; ^5^ Centre for Brain and Cognitive Development Department of Psychological Sciences Birkbeck, University of London London UK

**Keywords:** Psychotic experiences, emotional and behaviour problems, childhood, adolescence, twin study

## Abstract

**Background:**

Childhood emotional and behaviour problems are antecedents for later psychopathology. This study investigated genetic and environmental influences shaping the longitudinal association between childhood emotional and behaviour problems and specific PEs.

**Method:**

In a community‐based twin sample, parents reported on emotional and behaviour problems when twins were ages 7 and 12 years. At age 16 years, specific PEs were measured using self‐reports and parent reports. Structural equation model‐fitting was conducted.

**Results:**

Childhood emotional and behaviour problems were significantly associated with paranoia, cognitive disorganisation and parent‐rated negative symptoms in adolescence (mean *r *= .15–.38), and to a lesser extent with hallucinations, grandiosity and anhedonia (mean *r* = .04‐.12). Genetic influences on childhood emotional and behaviour problems explained significant proportions of variance in adolescent paranoia (4%), cognitive disorganisation (8%) and parent‐rated negative symptoms (3%). Unique environmental influences on childhood emotional and behaviour problems explained ≤1% of variance in PEs. Common environmental influences were only relevant for the relationship between childhood emotional and behaviour problems and parent‐rated negative symptoms (explaining 28% of variance) and are partly due to correlated rater effects.

**Conclusions:**

Childhood emotional and behaviour problems are significantly, if weakly, associated with adolescent PEs. These associations are driven in part by common genetic influences underlying both emotional and behaviour problems and PEs. However, psychotic experiences in adolescence are largely influenced by genetic and environmental factors that are independent of general childhood emotional and behaviour problems, suggesting they are not merely an extension of childhood emotional and behaviour problems.

## Introduction

Psychotic experiences (PEs, sometimes referred to as psychotic‐like experiences), such as paranoia and hallucinations, are traits within the general population that at the extreme are characteristic of symptoms of psychotic disorders such as schizophrenia (Wigman et al., 2011). Individuals with PEs are at an increased risk of developing emotional and psychotic disorders (McGrath et al., 2016), particularly if PEs persist over time (Dominguez, Wichers, Lieb, Wittchen, & van Os, 2011). PEs in mid to late adolescence occur just prior to the peak age of onset of many psychiatric conditions, such as psychotic disorder, depression and bipolar disorders (Laursen, Munk‐Olsen, Nordentoft, & Bo Mortensen, 2007). Thus, identifying early correlates of PEs could be useful for identifying antecedents of later psychiatric disorders.

Child and adolescent psychopathology is an established risk factor for a wide range of mental health conditions in later life (Copeland, Shanahan, Costello, & Angold, 2011). Emotional and behaviour problems in youth can precede anxiety, mood and disruptive disorders in later life (Reef, van Meurs, Verhulst, & van der Ende, 2010). Emotional and behaviour problems increase the risk of developing generalised anxiety disorder, major depressive disorder (Moffitt et al., 2007), nonaffective psychosis (Welham et al., 2009) and schizophreniform disorder (Kim‐Cohen et al., 2003). Similarly, associations have been observed between diagnosed psychiatric disorders in childhood, such as oppositional/conduct disorder, and later PEs (Siebald, Khandaker, Zammit, Lewis, & Jones, 2016). Furthermore, PEs often co‐occur with other psychopathologies such as depression (Freeman et al., 2012) which are often preceded by earlier signs of emotional and behaviour problems (Goodwin, Fergusson, & Horwood, 2004). Thus, it is possible that emotional and behaviour problems also share an underlying aetiology with PEs. Studies have found that youth who reported PEs, as defined by delusions and hallucinations, showed increased levels of internalising problems (Scott et al., 2009) and externalising problems (Polanczyk, Moffitt, & Arseneault, 2010). Emotional and behaviour problems in youth may form part of a trajectory for the development of later PEs (although it is acknowledged that some associations may be bidirectional; McGrath et al., 2016). Traditionally, externalising and internalising behaviours (which are captured here by general emotional and behaviour problems) are distinct components to thought disorder in principal component analyses, although a recent study reported a single ‘p‐factor’ – a single general psychopathology dimension capturing externalising, internalising and thought disorder symptoms (Caspi et al., 2014). Little is known about the underlying mechanisms that contribute towards the risk of developing both general childhood emotional and behaviour problems and later PEs.

One potential mechanism is shared genetic propensity. Emotional and behaviour problems (33%–81%) (Saudino, Ronald, & Plomin, 2005) and PEs (33%–58%) (Ronald, 2015) are in part heritable, thus making it possible that the two may covary because of common genetic influences. For example, there are several putative neurotransmitter systems that may be shared by both emotional and behaviour problems and PEs, such as the dopaminergic system. This system is a potential pathway through which pleiotropic genetic factors may have their influence. A recent twin study reported shared genetic influences between auditory hallucinations and a neuropsychiatric factor comprising ADHD, tic disorder, developmental coordination disorder and learning difficulties (Cederlöf et al., 2016). Using measured genotypes, recent research on singletons has reported significant genetic overlap between clinically recognised psychiatric disorders (Bulik‐Sullivan et al., 2015).

Emotional and behaviour problems and PEs also share similar environmental risk factors such as bullying (Arseneault, Bowes, & Shakoor, 2010; Schreier et al., 2009; Shakoor et al., 2015). It is possible that the same environmental risk factors lead emotional and behaviour problems and PEs to co‐occur. For example, the relationship between bullying and depression is moderated by cognitive vulnerabilities such as brooding (Garnefski & Kraaij, 2014). Similarly, cognitive vulnerabilities have been identified to increase the risk for developing PEs (Garety, Kuipers, Fowler, Freeman, & Bebbington, 2001). It is possible that exposure to environmental stressors such as bullying can contribute towards distortions in cognitive processes which in turn increase the risk of developing emotional and behaviour problems and PEs.

To our knowledge, no other study has investigated the associations between childhood emotional and behaviour problems (covering internalising and externalising behaviours and peer problems) and specific PEs (including positive, cognitive and negative experiences) in adolescence or estimated the extent to which genetic and environmental influences contribute to these relationships. Our aims were thus to examine if childhood emotional and behaviour problems are associated with specific PEs in adolescence, and to estimate the extent to which genetic and environmental factors influence the association between childhood emotional and behaviour problems and adolescent PEs.

## Methods

### Sample

Participants came from the Twins Early Development Study (TEDS), a sample of monozygotic (MZ) and dizygotic (DZ) twins born in England and Wales between 1994 and 1996 and assessed longitudinally across childhood and adolescence (Haworth, Davis, & Plomin, 2013). Participants were contacted at ages 7 and 12 to participate as a part of the TEDS study and then again at age 16, as a part of the Longitudinal Experiences And Perceptions (LEAP) study, which focused on PEs in adolescence (Ronald et al., 2014). TEDS and LEAP have full ethical approval and written consent was obtained at points of contact. Participants were excluded from the analyses if they did not provide consent at first contact (when TEDS was started), if they had severe medical disorder, had experienced severe perinatal complications, or if their zygosity was unknown. Data on emotional and behaviour problems at 7 and 12 years were available for a total of 5,537 and 4,953 families. Participating children had a mean age of 7.06 years and 11.56 years. At age 16, data on PEs were available for 5,076 families (mean age 16.32 years). Missing data were handled using full information maximum (Baraldi & Enders, 2010).

### Measures

#### Emotional and behaviour problems

Parents completed the Strengths and Difficulties Questionnaire (Goodman, 1997) when the twins were ages 7 and 12 years. Twenty items forming the Hyperactivity, Conduct, Peer Problem and Emotional problems subscales were summed to create a composite Strengths and Difficulties Questionnaire (SDQ) difficulties score, as per the published instrument; items assessing prosocial behaviour were excluded, as per the published instrument. This total difficulties score was used as a measure of emotional and behaviour problems.

#### Psychotic experiences

Psychotic experiences were assessed using the Specific Psychotic Experiences Questionnaire (SPEQ) (Ronald et al., 2014). SPEQ assesses specific PEs as quantitative traits and includes five self‐report subscales: paranoia (15 items – for example, ‘Someone has bad intentions towards me’), hallucinations (nine items – for example, ‘Hear noises or sounds when there is nothing about to explain them’), cognitive disorganisation (11 items – for example, ‘Often have difficulties in controlling your thoughts’), grandiosity (eight items – for example, ‘I have a special mission’), anhedonia (10 items – for example, ‘I don't look forward to things like eating out at restaurants’) and one parent‐rated subscale: parent‐rated negative symptoms (10 items – for example, ‘Has very few interests or hobbies’). SPEQ items were derived for the most part from existing scales that were adapted to be suitable for adolescents. The subscales were derived from principal component analysis that produced a scree plot suggesting a six‐component solution explaining 44.6% of the variance. The subscales showed good‐to‐excellent internal consistency (Cronbach's α = .77–.93) and test–retest reliability across a 9‐month interval (*r* = .65–.74) in this sample. Expert clinical opinion was obtained on the suitability of each item as a measure of adolescent psychotic experiences to ensure content validity (Ronald et al., 2014). For all SPEQ subscales except anhedonia, individuals who reported a family history (having a first‐ or second‐degree relative with schizophrenia or bipolar disorder) scored higher than individuals without a family history (all *p* < .05 except hallucinations, which showed a trend) (Zavos et al., 2014). Levels of agreement between scores on SPEQ and the PLIKS (an established measure of psychosis‐like symptoms) (Zammit, Owen, Evans, Heron, & Lewis, 2011) have been used to validate the SPEQ subscales (Ronald et al., 2014).

### Statistical analyses

Analyses were performed using STATA 12 (StataCorp, 2011) and OpenMx (Boker et al., 2011). OpenMx uses the method of full maximum likelihood estimation and is widely used for analysing genetically sensitive data. In line with standard behavioural genetics procedure, the effects of sex and age were regressed out as similarity due to age and sex can result in increasing phenotypic similarity within twin pairs, thus analyses were conducted using standardised residuals (Eaves, Eysenck, & Martin, 1989). Scales of emotional and behaviour problems and PEs were transformed using square root transformation techniques to reduce skewness and kurtosis and to ensure that the assumption of having a normal distribution was met for genetic modelling.

The twin design involves comparing within‐pair similarities of MZ and DZ twin pairs to determine the extent to which variation in a single phenotype, or covariation between phenotypes, is attributable to genetic and environmental influences. For a detailed explanation of the twin model please see Plomin, Defries, Knopik, & Neiderhiser, 2013. Structural equation modelling techniques were employed to establish the relative importance of additive genetic (A), common environment (C) and unique environmental influences (E) contributing to a phenotype (Plomin et al., 2013).

We used multivariate Cholesky decomposition to decompose the covariance between emotional and behaviour problems at age 7 and 12 and PEs at age 16 because this decomposition allowed for the utilisation of the longitudinal data. The Cholesky decomposition assesses the extent to which genetic and environmental influences underlying one trait also influence the others in the model (Neale & Cardon, 1992). It allows for the estimation of genetic influences contributing to the covariance between emotional and behaviour problems at age 12 and PEs independent of emotional and behaviour problems at age 7, and emotional and behaviour problems at age 7 and PEs independent of emotional and behaviour problems at age 12. Furthermore, the Cholesky decomposition enabled genetic influences to be estimated for PEs that were independent of those shared with emotional and behaviour problems at age 7 and 12.

The relative fit of different models were compared to a saturated model (which provides a full description of the data) to establish the best fitting model for the data (Rijsdijk & Sham, 2002). The best fitting models were selected on the basis of goodness of fit using the likelihood ratio test and the Bayesian Information Criterion (BIC), where lower BIC values indicated a better fit.

Phenotypic analyses – the association between emotional and behaviour problems and PEs was assessed using Pearson's correlations. To estimate the impact of emotional and behaviour problems we calculated population attributable fractions (PAF), which assumes a direct causal relationship between childhood emotional & behavioural problems and PEs. This was calculated using maximum likelihood estimations from logistic models (Greenland & Drescher, 1993). In addition, sensitivity, specificity, positive predictive values (PPV) and negative predictive values (NPV) were calculated as indices of risk for PEs among those with emotional and behavioural problems. To estimate these statistics, emotional and behaviour problems scores were dichotomised using the published cut‐off score of 17‐40 (Goodman, 1997). PEs scores were dichotomised using approximately the top 7% of the sample distribution to match reported prevalence (Linscott & Van Os, 2013). All other analyses employed the data as quantitative scales.

Genetic analyses — for emotional and behaviour problems and PEs that showed a mean phenotypic correlation ≥0.15 across the two ages of emotional and behaviour problems, similarity within twin pairs was calculated using intraclass correlations separately for MZ and DZ groups. Univariate structural equation models were used to estimate the contributions of genetic and environmental influences on emotional and behaviour problems and specific PEs. Multivariate Cholesky decompositions were performed to test (a) the degree to which genetic and environmental influences explained the association between emotional and behaviour problems and PEs, and (b) the proportion of genetic and environmental influences on PEs independent of those shared with emotional and behaviour problems. Sources of covariation were tested using the full ACE model, followed by the CE and AE models. This was followed by testing models where covariance paths were dropped between emotional and behaviour problems and PEs.

## Results

### Phenotypic analyses

Analysis of variance showed significant mean effects of sex on PEs (Table 1). Females reported higher levels of paranoia, hallucinations and cognitive disorganisation, in contrast to males who reported higher levels of grandiosity, anhedonia and had more parent‐rated negative symptoms (*p *<* *.05). Males also had higher levels of emotional and behaviour problems at age 7 and 12 than females (*p *<* *.05). A main effect for zygosity was observed for paranoia, hallucinations, cognitive disorganisation and parent‐rated negative symptoms, whereby DZs reported higher levels in comparison to MZs. The combined effect of gender and zygosity on the means was small (*R* ^2^ = 0.00–0.06).

**Table 1:** Means, standard deviations and analysis of variance by sex and zygosity for psychotic experiences and emotional and behaviour problems

	Total	Male	Female	MZ	DZ	Score range	Skew	Kurtosis	Cronbach *α*	ANOVA				
	*M* (SD)	*M* (SD)	*M* (SD)	*M* (SD)	*M* (SD)					Sex	Zyg	Sex*Zyg	*R* ^2^	*N*
Psychotic experiences														
Paranoia	12.17 (10.62)	11.76 (10.43)	12.50 (10.77)	11.79 (10.45)	12.38 (10.71)	0–72	0.18	3.05	0.93	<0.01	0.01	0.47	0.00	4,798
Hallucination	4.66 (6.02)	4.30 (5.77)	4.95 (6.19)	4.46 (5.91)	4.77 (6.07)	0–45	0.53	2.69	0.87	<0.01	0.01	0.55	0.01	4,806
Cognitive disorganisation	3.96 (2.85)	3.40 (2.72)	4.41 (2.87)	3.85 (2.82)	4.02 (2.86)	0–11	0.44	2.35	0.73	<0.01	0.01	0.58	0.03	4,799
Grandiosity	5.32 (4.43)	5.82 (4.57)	4.91 (4.26)	5.26 (4.35)	5.35 (4.47)	0–24	‐0.10	2.79	0.85	<0.01	0.56	0.98	0.01	4,802
Anhedonia	17.33 (7.93)	19.50 (8.00)	15.59 (7.44)	17.09 (7.97)	17.46 (7.91)	0–50	0.49	3.04	0.78	<0.01	0.58	0.80	0.06	4,802
Parent‐rated negative symptoms	2.81 (3.88)	3.17 (4.09)	2.51 (3.67)	2.63 (3.56)	2.91 (4.05)	0–30	0.60	2.78	0.85	<0.01	0.04	0.02	0.01	4,817
Emotional and behaviour problems: Parent report														
Age 7	8.12 (4.86)	8.63 (4.92)	7.67 (4.76)	7.95 (4.73)	8.22 (4.93)	0–30	−0.11	3.16	0.78	<0.01	0.12	0.58	0.01	5,537
Age 12	6.81 (4.90)	7.51 (5.08)	6.20 (4.66)	6.65 (4.79)	6.90 (4.96)	0–30	−0.10	3.15	0.81	<0.01	0.29	0.93	0.02	4,953

Means and standard deviation reported prior to transformation. Skew and kurtosis reported after transformation for normality. MZ = monozygotic, DZ = dizygotic twins. Analyses of variances were performed using one random member of each twin pair. Sex = *p*‐value associated with the effect of sex on the means; Zyg. = *p*‐value associated with the effect of zygosity on the means; Sex*Zyg = *p*‐value associated with the effects of the interaction between sex and zygosity on the means; *R* ^2^ = proportion of the total variance explained by sex and zygosity; *N* = number of randomly selected individuals from each twin pair.

Emotional and behaviour problems at ages 7 and 12 were moderately associated with paranoia (age 7 *r* = .13, age 12 *r* = .17, all *p *<* *.001), cognitive disorganisation (age 7 *r*= .16, age 12 *r* = .22, all *p *<* *.001), parent‐rated negative symptoms (age 7 *r* = .34, age 12 *r *= .42, all *p *<* *.001), and to a lesser extent with hallucinations (age 7 *r* = .10, age 12 *r* = .13, all *p *<* *.001), grandiosity (age 7 *r* = .04 *p *<* *.05, age 12 *r* = .03 *p *>* *.05) and anhedonia (age 7 *r* = .06, age 12 *r* = .12, all *p *<* *.001) (*See* Tables S1 and S7). We did not perform behaviour genetic analysis on hallucinations, grandiosity and anhedonia with emotional and behaviour problems, because phenotypic correlations were on average <.15 and therefore considered too small to be decomposed into genetic and environmental influences.

The PAF of adolescents with PEs for those with emotional and behaviour problems was low (*See* Table S2). PAF was significant for paranoia (age 7 PAF = 6%, age 12 PAF = 10%, all *p *<* *.05), cognitive disorganisation (age 7 PAF = 6%, age 12 PAF = 8%, all *p *<* *.05) and parent‐rated negative symptoms (age 7 PAF = 15%, age 12 PAF = 17%, all *p *<* *.05). PAF was not significant for hallucinations (with exception of age 12), grandiosity and anhedonia. These PAF estimates ranged from 1%–3% (*See* Table S2).

Overall sensitivity estimates were low (6%–21%) and specificity estimates were high (94%–96%), suggesting that having emotional and behavioural problems were only associated with PEs in 6%–21% of adolescents and that most without emotional and behavioural problem did not develop PEs. Positive predictive values indicated that a small to modest proportion (7%–26%) of individuals with emotional and behavioural problems went on to have PEs in later adolescence (See Table S3).

### Twin model‐fitting analyses

For emotional and behaviour problems, paranoia, cognitive disorganisation and parent‐rated negative symptoms, univariate twin correlations (*See* Table S4) were indicative of genetic influences (A), because MZ correlations were consistently larger than DZ correlations. DZ correlations were greater than half of MZ correlations with the exception of cognitive disorganisation, suggesting some common environmental (C) influence on paranoia, parent‐rated negative symptoms, and childhood emotional and behaviour problems. MZ correlations were less than unity and implied a moderate unique environmental effect (E) on all of the scales.

Univariate model‐fitting analyses showed genetic (A = 0.45–0.57) and unique environmental (E = 0.17–0.55) influences contributed to variances in PEs (*See* Table S5). A small proportion of the variance for parent‐rated negative symptoms was explained by common environment (C = 0.26). Common environmental parameters could be dropped from the models for paranoia and cognitive disorganisation. Genetic factors (A = 0.50–0.63) explained the largest proportions of variance in emotional and behaviour problems, followed by modest proportions explained by unique environmental influences (E = 0.20–0.25). Common environmental factors explained less of the variance in emotional and behaviour problems at age 7 in comparison to age 12 (C = 0.12 and C = 0.30 respectively) (*See* Table S5). All univariate ACE models did not provide a significantly worse fit compared to the saturated model.

**Figure 1:**
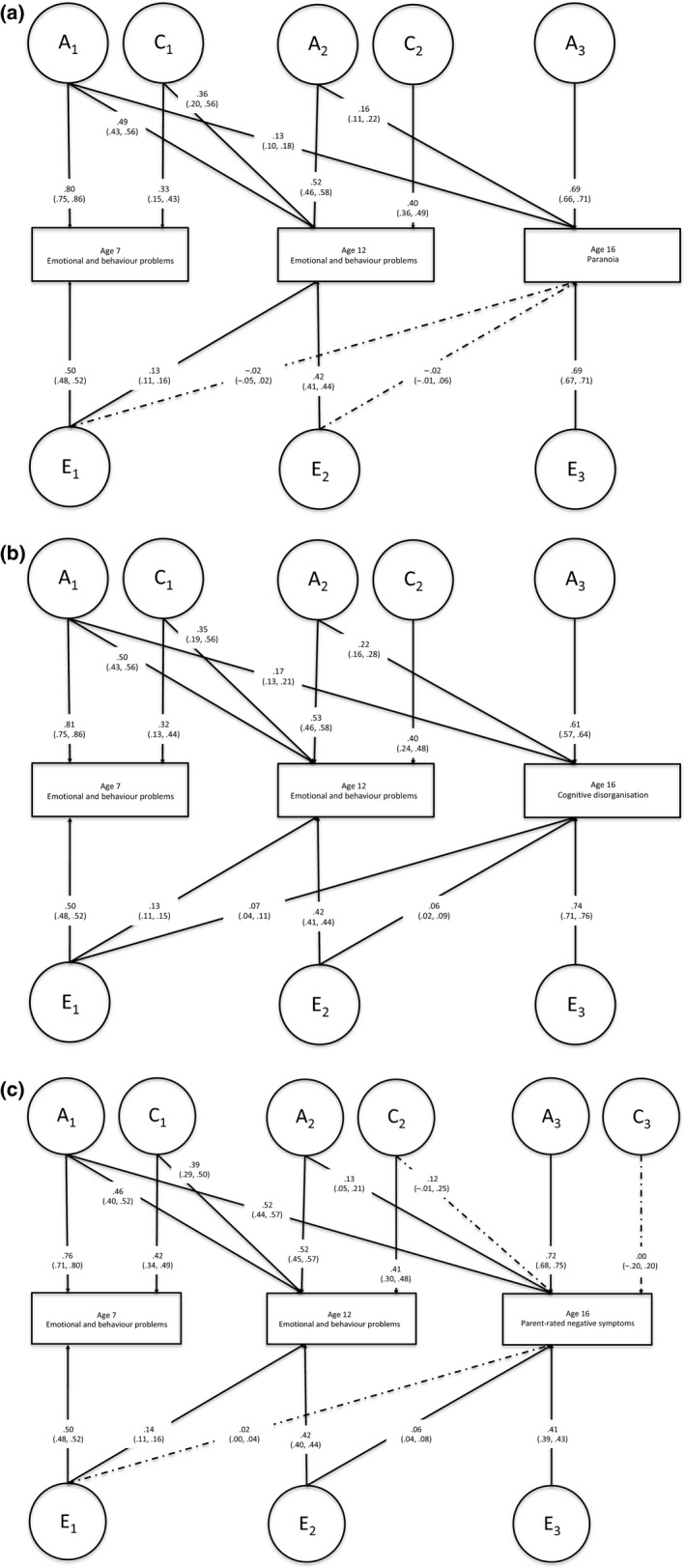
(a) Cholesky decomposition with emotional and behaviour problems at age 7 and 12 and paranoia at age 16. Standardised unsquared path coefficients and confidence intervals are shown. (b) Cholesky decomposition with emotional and behaviour problems at age 7 and 12 and cognitive disorganisation at age 16. Standardised unsquared path coefficients and confidence intervals are shown. (c) Cholesky decomposition with emotional and behaviour problems at age 7 and 12 and parent‐rated negative symptoms at age‐16. Standardised unsquared path coefficients and confidence intervals are shown. A = genetic effects, C = common environmental effects and E = unique environmental effects

Multivariate Cholesky decomposition showed that for the association between emotional and behaviour problems and paranoia, the ACE model with dropped cov_c_ with paranoia fitted the data best based on the likelihood ratio test and BIC fit index (*See* Table S6). Analyses demonstrated that approximately 4% (0.13^2^ + 0.16^2^ = 0.04) of the variance in paranoia was explained by emotional and behaviour problems at age 7 and 12 via genetic factors (comprising 1.7% from age 7 and 2.6% from age 12) (Figure 1a). Emotional and behaviour problems at age‐7 and 12 did not contribute to the total variance in paranoia via unique environmental influences (−0.02^2^ + 0.02^2^ = 0.00) (Figure 1a).

The relationship between emotional and behaviour problems and cognitive disorganisation had a similar pattern whereby the ACE model with dropped cov_c_ fitted the data best (*See* Table S6). Approximately 8% variance in cognitive disorganisation was explained by emotional and behaviour problems at age 7 and 12 via genetic factors (comprising 2.9% from age 7 and 4.8% from age 12) (Figure 1b). One per cent of the total variance in cognitive disorganisation was explained by emotional and behaviour problems at age 7 and age 12 via unique environmental influences.

The full ACE model fitted the data the best when investigating the association between emotional and behaviour problems and parent‐rated negative symptoms. Approximately 3% variance in parent‐rated negative symptoms was explained by emotional and behaviour problems at ages 7 and 12 via genetic factors (comprising 1.4% from age 7 and 1.7% from age 12) (Figure 1c). Twenty‐eight per cent of the variance in parent‐rated negative symptoms was explained by emotional and behaviour problems at age 7 and 12 via common environmental influences. Emotional and behaviour problems at age 7 and 12 did not contribute significantly to the total variance in parent‐rated negative symptoms via unique environmental influences.

## Discussion

Emotional and behaviour problems in childhood are a significant correlate of specific psychotic experiences (PEs) in adolescence. Extending previous studies reporting an association between emotional and behaviour problems and PEs (Polanczyk et al., 2010; Scott et al., 2009), this study found emotional and behaviour problems in childhood to be associated with a wider variety of specific PEs, with varying magnitude of effect. There was a trend for emotional and behaviour problems in childhood to be associated with higher levels of paranoia, cognitive disorganisation and parent‐rated negative symptoms, and to a lesser extent with more hallucinations, grandiosity and anhedonia at age 16. Associations with adolescent PEs were significant for emotional and behaviour problems assessed in children as young as age 7 years. The modest phenotypic associations were mirrored in the PAF and positive predictive values, which suggest that 6%–21% of adolescents with high PEs scores (paranoia, cognitive disorganisation and parent‐rated negative symptoms) were associated with earlier emotional and behaviour problems.

Our genetically sensitive twin design showed that small but significant proportions of variance in paranoia, cognitive disorganisation and parent‐rated negative symptoms was shared with childhood emotional and behaviour problems via common genetic influences. Similar proportions of genetic variance were carried over from age 7 and 12 emotional and behaviour problems (e.g. 1.7% of the genetic variance in paranoia was shared with age 7 emotional and behaviour problems and 2.6% with age 12 emotional and behaviour problems). Findings show that genetic predispositions that contribute towards children's vulnerabilities to having emotional and behaviour problems as early as age 7 years are shared with their predisposition for PEs at age 16 years. Identifying individuals with a family history of mental health conditions or a known genetic risk are strategies to help support those at risk of developing PEs. Certainly our results suggest that genetic predisposition for emotional and behavioural problems in childhood would not be a strong predictor of vulnerabilities for developing PEs during adolescence. These results suggest that a strategy of early detection and intervention with childhood behaviour problems would not be widely effective for preventing later psychotic experiences. At best, one in six cases of negative symptoms in midadolescence, and 1 in 20 cases of paranoia or cognitive disorganisation (case being used here to describe scorers in the highest 7% of the distribution) may be prevented assuming that there is a direct causal relation and the intervention is 100% effective.

In the same way that genetic overlap has been reported between distinct categories of adult psychiatric disorders (such as schizophrenia and major depressive disorder) (Lee et al., 2013), genetic overlap, albeit modest, is apparent in our study between conceptually distinct psychopathology traits in childhood and adolescence (emotional and behaviour problems and later PEs). A minority of genetic influences on emotional and behaviour problems in youth also explain PEs in adolescence. If this proportion was larger one could conclude that aetiologically, overlapping genetic influences explains the development of both emotional and behaviour problems and PEs. However, as the genetic overlap in our study is modest this suggests that although there is some common genetic influence, there are also genetic influences that are independent and specific to preadolescent emotional and behaviour problems and adolescent PEs. The suggestion of a degree of nonoverlapping causal processes in childhood and adolescent psychopathology has parallels with other research. For example childhood‐onset and adolescence‐onset antisocial behaviour may be aetiologically distinct (Silberg, Moore, & Rutter, 2015), and also findings from cognitive neuroscience show that the brain has structural and functional development in adolescence that is distinct from brain development during childhood (Blakemore, 2012). This warrants further investigation through molecular genetic studies.

Common environmental influences on emotional and behavioural problems explained part of the variance in parent‐rated Negative Symptoms but not the other types of PEs. Some of the same sociodemographic environmental factors are associated with both clinical psychiatric conditions and PEs as traits in the general population (Breetvelt et al., 2010). Parent reports were used to collect information about emotional and behaviour problems and negative symptoms dimension of PEs. Caution is warranted here since the higher common environmental estimates observed for the relationship between childhood emotional and behaviour problems and parent‐rated negative symptoms is likely inflated due to correlated rater effects, whereby the higher association is a product of both emotional and behaviour problems and parent‐rated negative symptoms being rated by the same individual (i.e. the parent).

Although our study has shown an overlap in genetic and environmental influences between general emotional and behaviour problems in childhood and some types of PEs in adolescence, it is important to note that a large proportion of genetic and environmental variance in adolescent PEs was independent of childhood emotional and behaviour problems. This suggests that new aetiological influences come into play in adolescence that influence the development of PEs independent of those associated with general emotional and behaviour problems in childhood. In terms of explaining the shared variance, a viable candidate for future research may be cognitive attribution styles, as cognitive biases have been associated with both emotional and behaviour problems and PEs (Garety et al., 2001; Zavos, Rijsdijk, Gregory, & Eley, 2010).

Our study has some limitations. Self‐reports were used to measure PEs (exception of parent‐rated negative symptoms). The inclusion of more data from multiple informants would have further strengthened our methodology. However, the SPEQ measure has shown good reliability and validity (Ronald et al., 2014). Measures of PEs were not available in early childhood, thus the effect of earlier PEs on the association between emotional and behaviour problems and PEs in adolescence could not be taken into account. Lastly, we studied a cohort of twins and we cannot be certain that our results generalise to singletons. However, the TEDS sample is representative of the United Kingdom (Haworth et al., 2013) and a similar mean value for emotional and behavioural problems (total SDQ score) has been observed between TEDs and samples of singletons (Meltzer, Gatward, Goodman, & Ford, 2000). This suggests that our findings are representative of the general population and may not be specific to twins.

This study also has a number of strengths. Using a genetically informative design, the longitudinal relationship between emotional and behaviour problems and later PEs was decomposed into genetic and environmental influences. Furthermore, this study included a wide array of specific PEs (positive, cognitive and negative PEs), which were measured as dimensions within a specific narrow age range. The large representative sample allowed the full spectrum of severity in psychopathology to be captured.

## Conclusion

Emotional and behaviour problems in childhood are associated with later PEs in adolescence. Although having emotional and behaviour problems as a child is linked with later PEs, they only explained small to modest proportions of variance in PEs. This suggests that other factors may be influencing onset of PEs in adolescence beyond those influencing childhood emotional and behaviour problems. Findings suggest that among individuals who experienced emotional and behaviour problems in childhood, the vulnerability for having later PEs may be due to genetically mediated factors. Future research into the specific genes that play a role in influencing both childhood emotional and behaviour problems and adolescent PEs is needed.

**Key Points**

- Emotional and behaviour problems in childhood were modestly associated with psychotic experiences in adolescence.
- Emotional and behaviour problems in childhood contributed to paranoia, cognitive disorganisation and parent‐rated negative symptoms in adolescents via genetically mediated mechanisms.
- Pleiotropic genetic effects, such as those known to operate between different categories of adult psychiatric illness, also appear to be present between different forms of psychopathology in childhood and adolescence.
- Psychotic experiences in adolescence are not merely an extension of childhood emotional and behaviour problems. They have genetic and environmental influences that are independent of general childhood emotional and behaviour problems.

## Supporting information


**Table S1.** Phenotypic correlations.
**Table S2.** Proportion of psychotic experiences attributable to emotional and behaviour problems.
**Table S3.** Sensitivity and specificity values, and positive and negative predictive values between emotional and behavioural problems at age 7 and 12 and psychotic experiences at age 16.
**Table S4.** Intraclass twin correlations.
**Table S5.** Fit statistics and parameter estimates for best fitting univariate models.
**Table S6.** Fit statistics for best fitting trivariate models.
**Table S7.** Interscale correlations between the subscales of emotional and behaviour problems.
