# Supplementary material for: A twin study exploring the association between childhood emotional and behaviour problems and specific psychotic experiences in a community sample of adolescents
Source: J Child Psychol Psychiatry. 2017 Nov 3;59(5):565–73. doi: 10.1111/jcpp.12839 (PMC5947637; doi:10.1111/jcpp.12839)
Supplement: Supplementary file 1 — Table S1. Phenotypic correlations. Table S2. Proportion of psychotic experiences attributable to emotional and behaviour problems. Table S3. Sensitivity and specificity values, and positive and negative predictive values between emotional and behavioural problems at age 7 and 12 and psychotic experiences at age 16. Table S4. Intraclass twin correlations. Table S5. Fit statistics and parameter estimates for best fitting univariate models. Table S6. Fit statistics for best fitting trivariate models. Table S7. Interscale correlations between the subscales of emotional and behaviour problems. [file JCPP-59-565-s001.docx]

Additional Supporting Information for: A twin study exploring the association between childhood emotional and behaviour problems and specific psychotic experiences in a community sample of adolescents – by S. Shakoor et al.

Table S1. Phenotypic correlations.

|  | Emotional and behaviour problems | |
| --- | --- | --- |
|  | Age-7 | Age-12 |
|  | r (CI) | r (CI) |
| Psychotic experiences | | |
| Paranoia | 0.13 (0.10, 0.16) | 0.17 (0.14, 0.20) |
| Hallucinations | 0.10 (0.07, 0.14) | 0.13 (0.10, 0.16) |
| Cognitive disorganisation | 0.16 (0.13, 0.19) | 0.22 (0.19, 0.25) |
| Grandiosity | 0.04 (0.01, 0.07) | 0.03 (-0.01, 0.06) |
| Anhedonia | 0.06 (0.03, 0.09) | 0.12 (0.08, 0.15) |
| Parent-rated negative symptoms | 0.34 (0.31, 0.37) | 0.42 (0.39, 0.45) |
|  | | |

Note: Correlations were performed using one random member of each twin pair using standardised age and sex regressed residuals. r = Pearson’s correlation, CI= confidence intervals.

Table S2. Proportion of psychotic experiences attributable to emotional and behaviour problems.

|  | Prevalence N (%) | | | Population attributable fraction (95% CI) | | |
| --- | --- | --- | --- | --- | --- | --- |
|  | Total | No | Yes | Age-7 | | Age-12 |
| Psychotic experiences |  |  |  |  | |  |
| Paranoia | 4,798 | 4,433 (92.39) | 365 (7.61) | 6% (2%-9%) | | 10% (6%-13%) |
| Hallucination | 4,806 | 4,501 (93.65) | 305 (6.35) | 1% (-2%-4%) | | 5% (1%-8%) |
| Cognitive disorganisation | 4,799 | 4,428 (92.27) | 371 (7.73) | 6% (3%-10%) | | 8% (4%-11%) |
| Grandiosity | 4,802 | 4,512 (93.96) | 290 (6.04) | 1% (-2%-5%) | | 3% (0%-72%) |
| Anhedonia | 4,802 | 4,480 (93.86) | 340 (6.14) | 1% (-3%-3%) | | 2% (-1%-5%) |
| Parent-rated negative symptoms | 4,817 | 4,510 (93.63) | 307 (6.37) | 15% (10%-20%) | | 17% (12%-22%) |
|  | | | | | | |
| Emotional and behaviour problems: Parent report | | | | | | |
| Age-7 | 5,537 | 5,197 (93.86) | 340 (6.14) | - | - | |
| Age -12 | 4,953 | 4,710 (95.09) | 243 (4.91) | - | - | |

Note: Population attributable fraction presented as a proportion.

Table S3. Sensitivity and specificity values, and positive and negative predictive values between emotional and behavioural problems at age 7 and 12 and psychotic experiences at age 16.

|  | Predictive values | | | | | | | |
| --- | --- | --- | --- | --- | --- | --- | --- | --- |
|  | Age 7 | | | | Age 12 | | | |
|  | Sensitivity  (95%CI) | Specificity  (95%CI) | PPV  (95%CI) | NPV  (95%CI) | Sensitivity  (95%CI) | Specificity  (95%CI) | PPV  (95%CI) | NPV  (95%CI) |
| PEs |  |  |  |  |  |  |  |  |
| Par | 11% (8%-15%) | 95% (94%-95%) | 14% (10%-20%) | 93% (92%- 94%) | 14% (11%-19%) | 96% (95%-97%) | 22% (17%-29%) | 93% (92%-94%) |
| Hal | 7% (4%-11%) | 94% (94%-95%) | 8%(5%-12% ) | 94% (93%-,95%) | 9% (6%-14%) | 95% (95%-96%) | 13% (8%-18%) | 94% (93%-95%) |
| Cog Dis | 12% (8%-16%) | 95% (94%-95%) | 16% (11%-21%) | 93% (92%, 94%) | 12% (9%-17%) | 96% (95%-96%) | 18% (13%-25%) | 93% (92%-94%) |
| Gran | 7% (4%-11%) | 94% (94%-95%) | 7% (4%-11%) | 94% (94%-95%) | 8% (5%-13%) | 95% (95%-96%) | 10% (6%-15%) | 94% (94%-95%) |
| Anhe | 6% (4%-10%) | 94% (94%-95%) | 7% (4%-11%) | 94% (93%-94%) | 7% (4%-11%) | 95% (95%-96%) | 10% (6%-15%) | 93% (92%-94%) |
| Par neg sym | 20% (15%-26%) | 95% (94%-96%) | 20% (15%-25%) | 95% (94%-96%) | 21% (16%-27%) | 96% (96%-97%) | 26% (20%-33%) | 95% (94%-96%) |

Note: PEs= Psychotic experiences, Par=paranoia, Hal=hallucinations, Cog Dis=cognitive disorganisation, Gran=grandiosity, Anhe= anhedonia, Par neg symp=parent-rated negative symptoms, PPV = positive predictive value, NPV =negative predictive value, CI= confidence intervals

Table S4. Intraclass twin correlations.

|  | MZ | DZ |
| --- | --- | --- |
|  | ICC (CI) | ICC (CI) |
| Univariate twin correlations | | |
| Psychotic experiences | | |
| Paranoia | 0.52 (0.49, 0.56) | 0.29 (0.24, 0.34) |
| Cognitive disorganisation | 0.45 (0.41, 0.48) | 0.23 (0.18, 0.28) |
| Parent-rated negative symptoms | 0.82 (0.81, 0.84) | 0.55 (0.52, 0.59) |
|  | | |
| Emotional and behaviour problems |  |  |
| Age-7 | 0.74 (0.72, 0.76) | 0.44 (0.40, 0.49) |
| Age-12 | 0.79 (0.77, 0.81) | 0.56 (0.52, 0.60) |
|  | | |
| Cross-trait cross-twin correlation | | |
| Psychotic experiences and Age-7 emotional and behaviour problems | | |
| Paranoia | 0.11 (0.06, 0.16) | 0.09 (0.03, 0.14) |
| Cognitive disorganisation | 0.10 (0.05, 0.15) | 0.06 (0.01, 0.12) |
| Parent-rated negative symptoms | 0.30 (0.25, 0.34) | 0.24 (0.19, 0.29) |
|  |  |  |
| Psychotic experiences and Age-12 emotional and behaviour problems | | |
| Paranoia | 0.16 (0.11, 0.21) | 0.10 (0.04, 0.15) |
| Cognitive disorganisation | 0.20 (0.15, 0.25) | 0.12 (0.07, 0.18) |
| Parent-rated negative symptoms | 0.37 (0.32, 0.41) | 0.29 (0.24, 0.34) |

Note: Intraclass correlations using transformed standardised age and sex regressed scales. ICC= Intraclass correlations CI= confidence intervals.

Table S5. Fit statistics and parameter estimates for best fitting univariate models.

|  |  | Model Fit | | | | | | | | |
| --- | --- | --- | --- | --- | --- | --- | --- | --- | --- | --- |
|  |  | Compared to saturated model | | | | | | Parameter estimates | | |
|  | Model | -2LL | df | LRT | Δdf | BIC | p | A (CI) | C (CI) | E (CI) |
| Paranoia | Sat | 23525.91 | 6527 | - | - | - | - | - | - | - |
|  | ACE | 23529.68 | 6533 | 3.77 | 6 | -29394.62 | .71 | .45 (.34, .54) | .07 (.00, .16) | .48 (.45, .52) |
|  | CE | 23598.74 | 6534 | 72.83 | 7 | -29333.66 | < 0.1 | - | - | - |
|  | *AE | 23531.56 | 6534 | 5.64 | 7 | -29400.84 | 0.58 | .52 (.49, .55) | - | .48 (.45, .51) |
| Cognitive disorganisation | Sat | 31571.04 | 6528 | - | - | - | - | - | - | - |
|  | ACE | 31580.54 | 6534 | 9.50 | 6 | -21351.86 | .15 | .44 (.32, .48) | .01 (.00, .11) | .55 (.52, .59) |
|  | CE | 31637.18 | 6535 | 66.14 | 7 | -21303.32 | <. 01 | - | - | - |
|  | *AE | 31580.58 | 6535 | 9.54 | 7 | -21359.92 | .22 | .45 (.42, .48) | - | .55 (.52, .58) |
| Parent-rated negative symptoms | Sat | 17410.51 | 6512 | - | - | - | - | - | - | - |
|  | *ACE | 17416.10 | 6518 | 5.59 | 6 | -35386.68 | .47 | .57 (.50, .64) | .26 (.19, .32) | .17 (.16, .18) |
|  | CE | 17810.00 | 6519 | 399.50 | 7 | -35000.89 | <. 01 | - | - | - |
|  | AE | 17465.87 | 6519 | 55.37 | 7 | -35345.02 | <. 01 | - | - | - |
| Emotional and behaviour problems:  Age-7 | Sat | 12936.75 | 5614 | - | - | - | - | - | - | - |
|  | *ACE | 12945.00 | 5620 | 8.25 | 6 | -34620.33 | .22 | .63 (.55, .72) | .12 (.03, .20) | .25 (.23, .27) |
|  | CE | 13191.07 | 5621 | 254.33 | 7 | -34382.72 | < .01 | - | - | - |
|  | AE | 12951.88 | 5621 | 15.14 | 7 | -34621.91 | < .01 | - | - | - |
| Emotional and behaviour problems:  Age-12 | Sat | 13282.75 | 5367 | - | - | - | - | - | - | - |
|  | *ACE | 13288.95 | 5373 | 6.20 | 6 | -32185.87 | .40 | .50 (.43, .58) | .30 (.23, .37) | .20 (.18, .22) |
|  | CE | 13515.26 | 5374 | 232.51 | 7 | -31968.03 | < .01 | - | - | - |
|  | AE | 13342.70 | 5374 | 59.95 | 7 | -32140.59 | < .01 | - | - | - |

Note: Sat = saturated model; -2LL = negative 2 log likelihood; df = degrees of freedom; LRT = likelihood ratio X^2^ test comparing the -2LL fit of each model to the -2LL fit of the saturated model; Δdf = difference in degrees of freedom comparing each model to the saturated model; BIC = Bayesian Information Criterion (lower values reflect a better fit); p = p-value. *Best fitting model.

Table S6. Fit statistics for best fitting trivariate models.

|  |  | Model Fit | | | | | | |
| --- | --- | --- | --- | --- | --- | --- | --- | --- |
|  |  | Compared to saturated model | | | | | | |
|  | Model | | -2LL | df | LRT | Δdf | BIC | p |
| Paranoia | Sat | | 43206.28 | 17526 | - | - | - | - |
|  | ACE | | 43230.33 | 17559 | 24.04 | 33 | -105381.70 | .87 |
|  | CE | | 43716.86 | 17565 | 510.58 | 39 | -104945.95 | <. 01 |
|  | AE | | 43295.45 | 17565 | 89.16 | 39 | -105367.36 | <. 01 |
|  | ACE dropped cov_a_ with Para | | 43305.56 | 17562 | 99.27 | 36 | -105331.86 | <. 01 |
|  | *ACE dropped cov_c_ with Para | | 43236.48 | 17562 | 30.20 | 36 | -105400.94 | . 74 |
| Cognitive disorganisation | Sat | | 43050.66 | 17527 | - | - | - | - |
|  | ACE | | 43085.40 | 17560 | 34.74 | 33 | -105535.09 | .39 |
|  | CE | | 43561.22 | 17566 | 510.56 | 39 | -105110.05 | <. 01 |
|  | AE | | 43148.26 | 17566 | 97.60 | 39 | -105523.01 | <. 01 |
|  | ACE dropped cov_a_ with Cog | | 43150.34 | 17563 | 99.68 | 36 | -105495.54 | < .01 |
|  | *ACE dropped cov_c_ with Cog | | 43092.20 | 17563 | 41.54 | 36 | -105553.68 | .24 |
| Parent-rated negative symptoms | Sat | | 40397.10 | 17513 | - | - | - | - |
|  | *ACE | | 40440.52 | 17546 | 43.42 | 33 | -108061.48 | .11 |
|  | CE | | 41250.76 | 17552 | 853.66 | 39 | -107302.02 | <. 01 |
|  | AE | | 40578.19 | 17552 | 181.09 | 39 | -107974.59 | <. 01 |
|  | ACE dropped cov_a_ with Neg | | 40834.58 | 17549 | 437.48 | 36 | -107692.81 | <. 01 |
|  | ACE dropped cov_c_ with Neg | | 40548.83 | 17549 | 151.73 | 36 | -107978.56 | <. 01 |

Note: Sat= Saturated model; ACE= Full model testing genetic, common and unique environmental influences; CE = Model testing common and unique environmental influences; AE = Model testing genetic and unique environment influences; ACE dropped cov_a_ = Model testing genetic, common and unique environmental influences with genetic covariances between all phenotypes dropped. ACE dropped cov_c_ = Model testing genetic, common and unique environmental influences with common environmental covariances between all phenotypes dropped. The same is applied to paranoia, cognitive disorganisation, and parent-rated negative symptoms. Para = paranoia, Cog = cognitive disorganisation, Neg = parent-rated negative symptoms. -2LL = negative 2 log likelihood; df = degrees of freedom; LRT = likelihood ratio X^2^ test comparing the -2LL fit of each model to the -2LL fit of the saturated model; Δdf = difference in degrees of freedom comparing each model to the saturated model; BIC = Bayesian Information Criterion (lower values reflect a better fit); p = p-value. *Best fitting model.

Table S7. Interscale correlations between the subscales of emotional and behaviour problems.

|  | **Paranoia**  r (CI) | **Hallucinations**  r (CI) | **Cognitive disorganisation**  r (CI) | **Grandiosity and delusion**  r (CI) | **Anhedonia**  r (CI) | **Parent-rated negative symptoms** r (CI) |
| --- | --- | --- | --- | --- | --- | --- |
| **Age 7** |  |  |  |  |  |  |
| Emotional and behaviour problems | 0.13 (0.10, 0.16) | 0.10 (0.07, 0.14) | 0.16 (0.13, 0.19) | 0.04 (0.01, 0.07) | 0.06 (0.03, 0.09) | 0.34 (0.31, 0.37) |
| Hyperactivity | 0.06 (0.03, 0.09) | 0.06 (0.03, 0.09) | 0.12 (0.09, 0.15) | 0.05 (0.02, 0.08) | 0.03 (-0.01, 0.06) | 0.25 (0.22, 0.28) |
| Conduct problems | 0.10 (0.06, 0.13) | 0.06 (0.03, 0.09) | 0.10 (0.07, 0.13) | 0.03 (-0.03, 0.06) | 0.06 (0.03, 0.09) | 0.23 (0.20, 0.26) |
| Peer problem | 0.12 (0.09, 0.15) | 0.08 (0.04, 0.11) | 0.08 (0.05, 0.11) | 0.02 (-0.01, 0.05) | 0.09 (0.06, 0.12) | 0.23 (0.20, 0.26) |
| Emotional problems | 0.09 (0.06, 0.12) | 0.07 (0.04, 0.10) | 0.13 (0.10, 0.16) | 0.01 (-0.03, 0.04) | 0.01 (-0.03. 0.04) | 0.20 (0.17, 0.23) |
| **Age12** |  |  |  |  |  |  |
| Emotional and behaviour problems | 0.17 (0.14, 0.20) | 0.13 (0.10, 0.16) | 0.22 (0.19, 0.25) | 0.03 (-0.01, 0.06) | 0.12 (0.08, 0.15) | 0.42 (0.39, 0.45) |
| Hyperactivity | 0.09 (0.06, 0.12) | 0.08 (0.05, 0.11) | 0.15 (0.11, 0.18) | 0.05 (0.02, 0.08) | 0.09 (0.06, 0.12) | 0.34 (0.31, 0.37) |
| Conduct problem | 0.12 (0.09, 0.15) | 0.09 (0.05, 0.12) | 0.12 (0.09, 0.15) | 0.02 (-0.01, 0.05) | 0.07 (0.04, 0.10) | 0.26 (0.24, 0.30) |
| Peer problems | 0.18 (0.14, 0.21) | 0.12 (0.09, 0.15) | 0.13 (0.09, 0.16) | 0.04 (0.01, 0.07) | 0.11 (0.09, 0.15) | 0.28 (0.25, 0.31) |
| Emotional problems | 0.11 (0.08, 0.15) | 0.11 (0.07, 0.14) | 0.20 (0.17, 0.23) | -0.02 (-0.06, 0.01) | 0.06 (0.03, 0.09) | 0.28 (0.25, 0.31) |

Note: Emotional and behaviour problems: Total score of SDQ scales
